# Examining the Efficacy of a 405 nm Wavelength Diode Laser as a Diagnostic Tool in Routine Dental Practice

**DOI:** 10.7759/cureus.62474

**Published:** 2024-06-16

**Authors:** Marwan El Mobader, Samir Nammour

**Affiliations:** 1 Department of Oral Surgery, Laser Laboratory, Wroclaw Medical University, Wroclaw, POL; 2 Department of Dental Sciences, Faculty of Medicine, University of Liege, Liege, BEL

**Keywords:** restorative dentistry, operative dentistry, periodontology, dental practitioner, oral diagnosis, dental lasers, laser dentistry, clinical dentistry

## Abstract

In recent years, significant advancements in dental and periodontal diagnostics have paved the way for improved care. Among the available approaches, laser fluorescence (LF) is a promising method. This case report explores the utilization of a 405 nm diode laser as a diagnostic tool in the non-surgical treatment of biofilm-induced gingivitis, as well as its application in routine daily practice for diagnosing restorations and dental caries. A 24-year-old male patient and a 21-year-old female patient were included. A 405 nm diode laser (Smart M, Lasotronix, Poland) was used as a diagnostic tool with a tip diameter of 8 mm. In case one, the utilization of the 405 nm diode laser enhanced the patient's ability to perceive the presence and extent of plaque and calculus, aiding in motivation and education regarding supra-gingival dental biofilm control and it assisted the operator in precisely localizing plaque and calculus, thereby enabling more effective mechanical debridement and ultimately improving treatment outcomes. In the second case, the utilization of the laser facilitated the detection of defective composite fillings aiding in both accurate diagnosis for the operator and effective communication with the patient regarding the need for re-treatment. This study illustrates the potential of the relatively new 405 nm diode laser as a promising diagnostic tool in the daily management of periodontal patients and the detection of defective dental fillings in daily practice.

## Introduction

Laser fluorescence (LF) has emerged as a promising diagnostic tool in dentistry [1]. This non-invasive technique utilizes specific wavelengths of light to assess the fluorescence properties of dental and periodontal tissues, aiding in the identification of demineralized areas indicative of caries formation and the presence of bacteria indicative of the presence of biofilm [2]. Additionally, LF can aid in monitoring lesion progression and evaluating the effectiveness of preventive measures, contributing to more personalized and proactive patient care [3,4]. Dental caries is a prevalent chronic condition characterized by the gradual breakdown of tooth structure. The traditional visual-tactile method, while widely used for early caries detection, has limitations [5]. It relies on observing changes in tooth color and texture using a dental explorer but exhibits low sensitivity and specificity, particularly for early detection [5]. Additionally, it may pose challenges in areas with limited visibility and could potentially exacerbate existing lesions [5]. Acknowledging these limitations, there is a growing interest in innovative approaches to caries detection [4,5]. LF has emerged as a promising alternative. Beyond its role in caries detection, LF has also shown promise in periodontal disease management [6]. By enabling the visualization and quantification of sub-gingival biofilm, LF can enhance the accuracy of plaque and calculus detection, supporting more effective periodontal therapy [6]. Periodontal diseases represent a multifactorial, biofilm-induced chronic inflammation that impacts the tissues supporting the teeth [7]. Given its high prevalence and significant negative impact on quality of life, particularly periodontitis, it has emerged as a major public health concern [7]. According to international clinical practice guidelines such as the ones suggested by the European Federation of Periodontology (EFP) and the American Academy of Periodontology (AAP), the gold standard treatment for periodontal diseases consists of the elimination of supra- and sub-gingival infections, through manual and/or ultrasonic instrumentation [8]. Hence, the key to the management of periodontal diseases remains to eliminate as much as possible the supra- and sub-gingival calculus and plaque which can lead to the resolution of the periodontal tissue’s inflammation and, hence, the reparation of the periodontium [8]. Adequate visualization and detection of biofilms are primordial for their elimination. Therefore, there is an ongoing need for improved methods of detecting plaque and calculus, particularly in areas that are not easily visualized [8,9]. Current practice in diagnosis in periodontology relies on subjective, qualitative visual assessment of the presence and abundance of the plaque and calculus. However, in other disciplines of medicine, such as in the management of chronic wounds, the use of bacterial LF showed positive and promising results in enhancing the overall diagnosis, thus, enhancing the overall management [10]. Since the use of 405 nm wavelength LF was proven to be effective in chronic wounds, and since the detection of a biofilm is the key to the treatment of periodontal diseases, a laser-diagnosis protocol was suggested in a patient with gingivitis. This case report aims to explore the effectiveness of LF diagnostic aids in the early detection of dental caries and the effectiveness of LF diagnostic at different steps of the non-surgical management of periodontal disease.

## Case presentation

Case 1

Presentation and Informed Consent

A 24-year-old male patient presented to the clinic for a routine check-up and scaling and root planning (SRP). The patient had no systemic disease that could affect his dental and periodontal health. During the clinical examination, maxillary and mandibular gingiva appeared swollen, exhibiting a bright reddish color and bleeding on probing (BoP). Moreover, plaque and calculus were detected in the supra- and subgingival area. The plaque index (PI) was calculated and a score of 3 was given. Moreover, BoP was calculated and a score of 80% was given. The diagnosis rendered was biofilm-induced gingivitis. Hence, non-surgical management of gingivitis based on the European Federation of Periodontology (EFP)’s clinical guidelines was suggested as first-line treatment. The treatment consists of three steps: step 1, supra-gingival dental biofilm control and motivational interviewing, step 2, non-surgical mechanical debridement, and step 3 consisting of a session of re-evaluation. The use of a 405 nm diode laser (Smart M, Lasotronix, Poland) for diagnosis was proposed to be integrated into each step. The aim of using the 405 nm diode laser (Smart M, Lasotronix, Poland) was explained to the patient. The patient agreed to the treatment and provided written informed consent before his enrollment. Intra-oral pictures were taken before and at different times of treatment.

Step 1. Supra-Gingival Dental Biofilm Control and Motivational Interviewing

**Figure 1:**
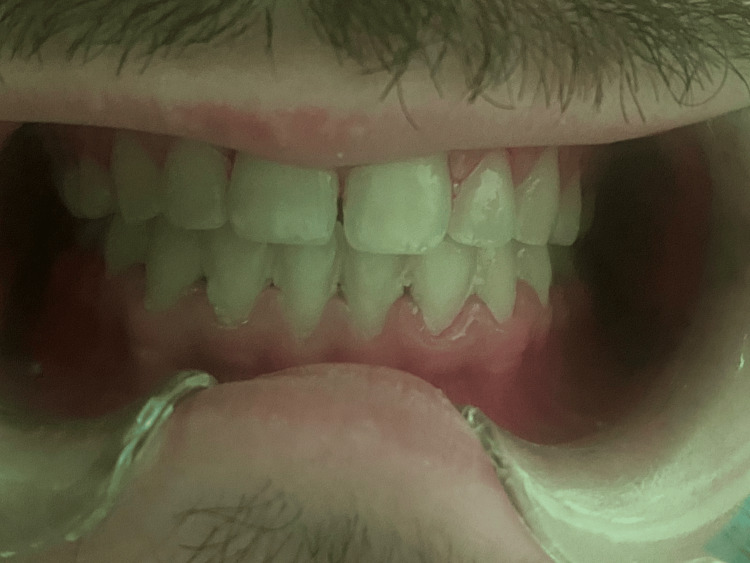
Aspect of the Mandibular Gingiva Before Treatment

**Figure 2:**
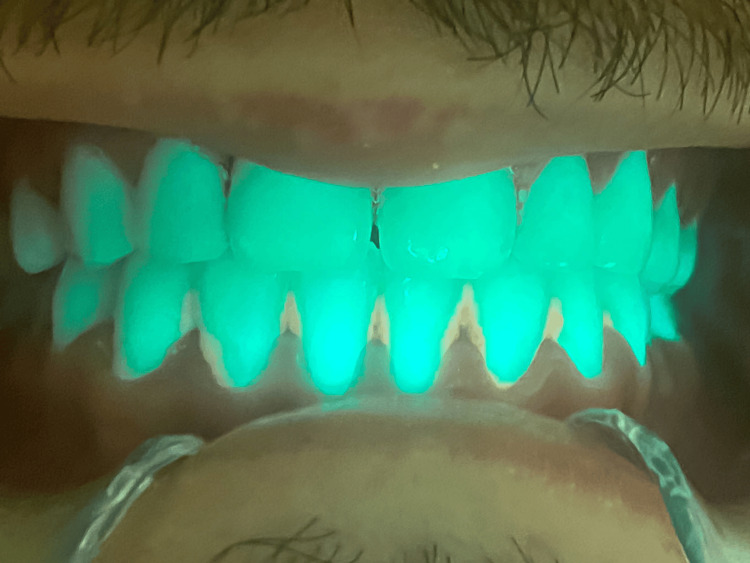
Aspect of the Mandibular Arch Under Laser Irradiation and Before Treatment: Enhanced Visualization of Plaque and Calculus Highlighted in Orange

During the first session with the patient, strategies for controlling risk factors that contribute to the formation of a biofilm were implemented. To address the principal risk factor, the accumulation of the biofilm as plaque and calculus, intra-oral photographs were taken without (Figure 1) and with the use of the 405 nm diode laser (Smart M, Lasotronix, Warsaw, Poland) (Figure 2). Irradiation parameters were as follows: non-contact mode with a distance of almost 15 cm, a tip diameter of 8 mm, continuous mode (CW), and a power of 320 mW/cm^2^. Scanning motion was made in each of the arches with a clockwise movement in order to have a proper diagnosis in each area. Irradiation time was not calculated as it was as much as needed to have a proper diagnosis. The irradiation parameters and protocol remained the same throughout all the steps of the treatment. As a result of using the 405 nm diode laser during step 1, the patient reported better visualization of the biofilm (plaque and calculus) when irradiation with the laser was made compared to non-irradiation. This helped the patient to locate the biofilm and to appreciate its abundance, hence, enhancing the supra-gingival dental biofilm control and motivation. After diagnosis, proper oral hygiene instructions were given to the patient. The oral hygiene instruction consisted of guidance on adopting an adequate tooth brushing technique (Bass technique) and using interdental brushes for interdental cleaning. The choice of the interdental brush was made based on the interdental area. 

Step 2. During Sub-Gingival Biofilm and Calculus Instrumentation

**Figure 3:**
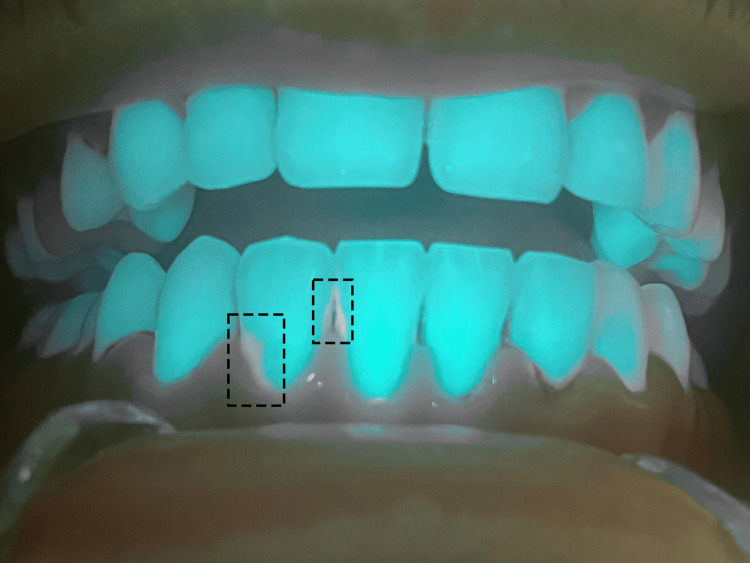
Aspect of the Mandibular Gingiva During Non-surgical Mechanical Debridement and Under Diode Laser Irradiation: Visualization of the Remaining Biofilm

After step 1, and at the same session, sub-gingival biofilm and calculus instrumentation was performed. The protocol consisted of professional sub-gingival plaque removal (SRP) using an ultrasonic piezoelectric scaler (Piezosteril 6, Castellini, Cazzago San Martino, Italy) for the entire mouth. Additionally, instrumentation with manual curettes (Universal and Gracey curettes) was conducted. Subsequently, chlorhexidine 0.12% solution (Eludril pro mouthwash, Pierre Fabre Oral Care, Paris, France) was used to irrigate the gingival sulcus. During instrumentation, the 405 nm wavelength laser was employed to examine for any remaining calculus or plaque post-treatment. A comparison between the appearance of the gingiva under direct observation and under laser irradiation demonstrated significantly enhanced visualization when the latter method was employed (Figure 3).

Step 3. Re-evaluation

**Figure 4:**
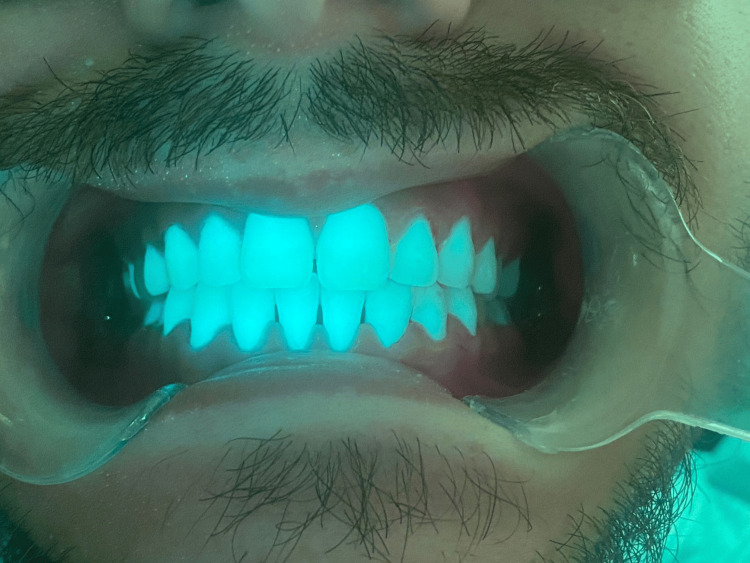
Aspect of the Mandibular Gingiva After Non-surgical Mechanical Debridement and Under Diode Laser Irradiation: Visualization of the Absence of the Biofilm

After a period of 10 days, a re-evaluation session was conducted to assess various parameters including plaque accumulation using the PI, BoP, and the overall condition of the gingiva. The PI was recorded as 2, while BoP was less than 30%. These scores indicated an improvement in oral hygiene and supra-gingival biofilm control. The 405 nm diode laser was employed during this stage to assess the presence or absence of biofilm. The irradiation confirmed the assessment of PI and BOP scores. The patient was invited to enhance his oral hygiene based on the illustration obtained from the laser irradiation. As per the operator's observations, the utilization of laser irradiation facilitated clearer identification of any residual biofilm and calculus during mechanical debridement. Following the detection of the residual biofilm, SRP procedures were conducted with emphasis on areas where the biofilm was apparent. Subsequently, laser irradiation was employed once more to ensure the complete removal of any remaining calculus and plaque. The use of the laser at the end of the mechanical debridement revealed a successful removal of the remaining biofilm (Figure 4).

Case 2

Case Presentation

A 21-year-old female patient presented to the clinic for a routine check-up and SRP. The patient did not have any systemic conditions that could affect her oral health. However, she did have irregular teeth and a crossbite, which could impede her overall oral hygiene. Upon clinical assessment, noticeable swelling and a bright red hue were observed in both the upper and lower gum tissues, which also bled upon probing. Additionally, deposits of plaque and calculus were found supra- and sub-gingival. The PI was determined, resulting in a score of 3. Furthermore, the percentage of bleeding upon probing was assessed, yielding a score of 60%. Biofilm-induced gingivitis was diagnosed. In addition to gingivitis, defective composite fillings were seen on the maxillary arch.

Irradiation Protocol

The identical laser-assisted diagnosis protocol from Case 1 was applied in Case 2 to detect the biofilm (plaque and calculus). Then, for the detection of the anterior composite fillings, the 405 nm diode laser (Smart M, Lasotronix, Poland) was used with the following parameters: continuous mode, non-contact mode with a distance of almost 15 cm, a power density of 160 mW/cm^2^, and a laser tip diameter of 8 mm. Scanning motion was made in each of the arches with a clock-wise movement in order to have a proper diagnosis in each area. The irradiation time was not calculated as it was as much as needed to have a proper diagnosis.

**Figure 5:**
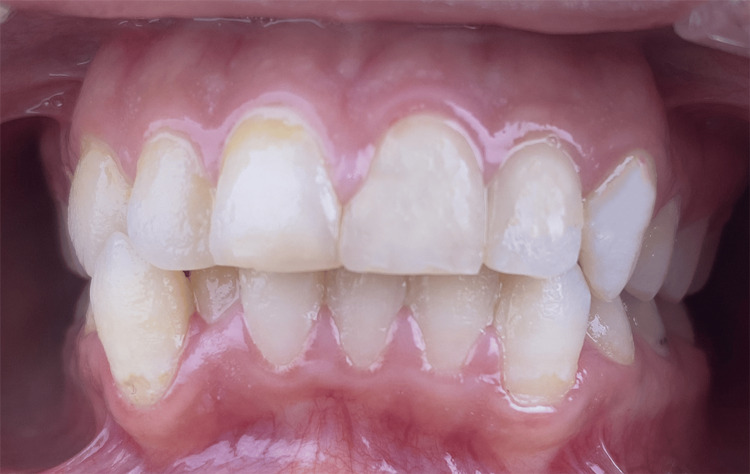
Aspect of the Oral Cavity Without Laser Irradiation

**Figure 6:**
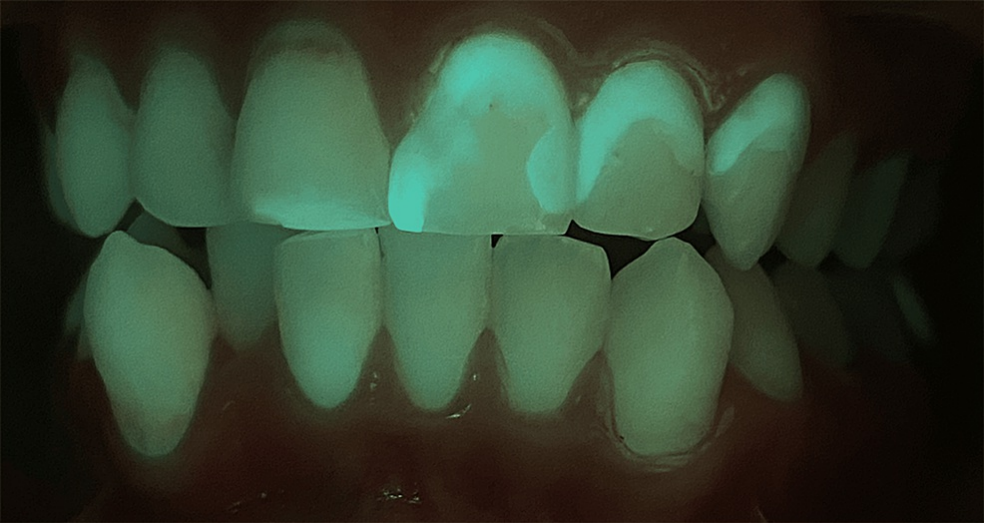
Enhanced Visualization of Composite Fillings During 405 nm Diode Laser Irradiation in the Oral Cavity

Intra-oral photographs were captured both without laser irradiation (Figure 5) and with laser irradiation (Figure 6) to compare biofilm detection. Both images revealed the presence of calculus and plaque; however, utilizing laser irradiation as a diagnostic tool resulted in a more vivid and highlighted visualization of the presence of defective composite fillings on the two central maxillary incisors, left maxillary lateral and left maxillary canine. The patient was able to detect and locate the defective composite fillings, which facilitated communication with her and enhanced her understanding of the importance of re-treatment. To treat the biofilm-induced gingivitis, the exact protocol that was applied in Case 1 was replicated in Case 2. Additionally, the defective composite fillings underwent re-treatment. This case report reveals the significant positive impact laser irradiation had on the proper detection of not only biofilm but also defective composite fillings.

## Discussion

There is a need in periodontal and dental care for innovative and scientifically validated diagnostic tools that can improve the management of periodontal and dental diseases. In fact, the ability to instantly and confidently identify the location of biofilms present in the form of calculus or plaque enables better communication with the patient and more precise debridement during scaling root planning with manual and ultrasonic instruments. Furthermore, precise identification of the presence, location, and extent of dental restorations facilitates appropriate treatment planning and enhances communication with patients. Hence, the objective of this study was to assess the advantages of integrating a diode laser (405 nm) as a diagnostic tool into the standard treatment protocol for biofilm-induced gingivitis and in the general daily practice in dentistry. In the management of gingivitis, the laser was employed throughout three pivotal stages of the treatment regimen: controlling supra-gingival dental biofilm and conducting motivational interviewing, performing mechanical debridement (scaling and root planning), and conducting re-evaluation sessions.

The utilization of the laser proved beneficial across the three phases of treatment. During the initial session, the patient noted enhanced clarity in visualizing supragingival biofilm with laser irradiation, facilitating improved understanding of abundance and location. Furthermore, during mechanical instrumentation, the operator experienced improved visualization of remaining calculus, particularly in interdental and subgingival locations. This enhanced visualization contributed to more effective mechanical debridement, theoretically leading to improved treatment outcomes. Additionally, defective composite fillings were readily discerned within the anterior sector during diagnostic procedures, aiding in the elucidation of the necessity for re-treatment. This clear visualization was advantageous for patient comprehension and informed decision-making regarding further treatment interventions. This study can be considered one of the rare attempts to utilize a diode laser (405 nm) for diagnosing and treating periodontal patients.

Despite the significance of detecting biofilms, the primary factor contributing to periodontal disease development, the literature lacks extensive reporting on the utilization of diagnostic tools in periodontal disease. A narrative study by Chang et al. revealed that periodontal endoscopy, optical coherence tomography, fluorescence spectroscopy, and differential reflectometry allow for a more accurate diagnosis of subgingival calculus deposits in comparison to detection via conventional periodontal probing using periodontal probes [11]. However, the study concluded that despite the improved results, longer operation times and expensive equipment are common limitations that need to be addressed. In this study, the utilization of the diode laser (405 nm) necessitates a relatively short operation time, as all parameters are preconfigured by the machine (Smart M, Lasotronix, Poland) requiring the operator solely to initiate the irradiation using the dedicated diagnostic tip. In parallel with our investigation, Qin et al. delineated a fluorescence-based methodology employing a 405 nm wavelength for real-time detection and quantification of calculus [12]. Qin et al. employed a 405 nm focused blue LED and demonstrated the capability of this technique to discern calculus from healthy teeth with 100% sensitivity and specificity. Their study shares similarities with our present research in employing a wavelength of 405 nm. Nevertheless, our protocol diverges from theirs as it does not necessitate specialized software, as described by Qin et al. [12]. Several limitations need to be addressed in our case report. Primarily, as a case report, no comparison with a control group was conducted, and statistical analyses were not performed. Secondly, unlike other studies, there was no quantitative assessment of the biofilm using methods such as PCR or TBC to correlate the visually obtained results with the actual bacterial count. However, it is crucial to regard this as an initial step for further exploration in the domain of periodontal management diagnosis. Consequently, additional research such as randomized clinical trials is necessary to facilitate the incorporation of these imaging and detection techniques into clinical settings.

## Conclusions

In these two cases, the utilization of a 405 nm diode laser as a diagnostic tool for managing biofilm-induced gingivitis aided in the detection and localization of supra- and sub-gingival calculus, as well as defective composite fillings. Its effectiveness was observed across the three primary stages of periodontal disease management. However, it is important to acknowledge the limitations of this case report. Nonetheless, this report serves as an initial step for further investigations into integrating diagnostic tools like the 405 nm diode laser into communication with periodontal patients, as well as the diagnosis and treatment of periodontal disease.
